# The National Cancer Institute R25 Cancer Education Grants Program: A Workshop Report

**DOI:** 10.1007/s13187-016-1161-8

**Published:** 2017-01-07

**Authors:** Jeannette F. Korczak, Davyd W. Chung, Erica Rosemond, Daniel D. Von Hoff, Richard L. Haspel, John W. Waterbor, Shine Chang, Amelie G. Ramirez, Susan Perkins, Jonathan Wiest, Ming Lei

**Affiliations:** 1 National Cancer Institute, 9609 Medical Center Dr., Room 2W110, Rockville, MD 20852 USA; 2 National Center for Advancing Translational Sciences, Bethesda, Maryland USA; 3 American Association for Cancer Research, Philadelphia, PA USA; 4 Beth Israel Deaconess Medical Center, Boston, MA USA; 5 University of Alabama at Birmingham School of Public Health, Birmingham, AL USA; 6 University of Texas MD Anderson Cancer Center, Houston, TX USA; 7 University of Texas Health Science Center at San Antonio, San Antonio, TX USA

**Keywords:** Cancer education, R25, NCI

## Abstract

Through the R25 Cancer Education Grants Program (CEGP), the National Cancer Institute (NCI) has been supporting the broad educational needs of the cancer research and cancer healthcare communities since 1974. NCI sponsored a workshop on September 13, 2016 in Bethesda, Maryland, with the objectives of sharing best practices in cancer education, communicating R25 CEGP programmatic information, and gathering ideas to strengthen the R25 CEGP to better meet the emerging needs in cancer education in the face of a rapidly changing landscape in cancer research and cancer care. With 53 leaders in cancer education in attendance, the workshop featured an overview of the R25 CEGP by NCI Program Staff, a showcase of several types of CEGP programs by current R25 grantees, and in-depth discussions on a broad range of questions critical for the continued success of the R25 CEGP. The workshop afforded an opportunity, for the first time, for cancer researchers and clinicians conducting different forms of cancer education activities to gather in one place as leaders of a community of increasing importance. The discussion resulted in a set of suggestions that will benefit the R25 CEGP and cancer education in general. There was a general consensus among the participants that bringing the cancer education community together is a significant achievement of the workshop that will have a long-lasting impact on cancer education.

**Electronic supplementary material:**

The online version of this article (doi:10.1007/s13187-016-1161-8) contains supplementary material, which is available to authorized users.

## Introduction

The National Institutes of Health (NIH) R25 research education program complements research training programs such as the National Research Service Awards (NRSA) and Career Development Awards (typically referred as K awards) in developing the nation’s current and future health research and healthcare workforce [1]. While the NRSA and K awards typically provide stipend/salary support to trainees in the early stages of their research careers for formal research training, R25 awards are designed to meet the diverse educational needs of a broad range of participants, including students, researchers, healthcare providers, and public health professionals. Typically, R25 awards provide funds to institutions for developing and implementing education programs that disseminate scientific discoveries, promote appreciation/interest in health research, offer hands-on exposure to biomedical research, and develop specific research skills in needed areas. To meet this broad set of goals, R25 programs make use of various forms of educational designs and approaches [1].

NCI began to support the Cancer Education Grants Program (CEGP) using the R25 funding mechanism in the wake of the initiation of the National Cancer Program authorized by the National Cancer Act of 1971 [2]. From 1974 to 1993, NCI solicited R25 grant applications for Professional Oncology Education Programs, Clinical Cancer Education Programs, and Cancer Education Programs through several Notices published in the NIH Guide [3–5]. When special educational needs arose, NCI also issued R25 Requests for Applications, e.g., for cancer education programs in pain management [6]. NCI-supported R25 projects during this period helped educate physicians, students, and patients about cancer and cancer research, as well as disseminate discoveries in new areas of cancer research and care. In 1993, NCI published its first formal R25 CEGP funding opportunity announcement (FOA) to provide institutions with a “wide range of opportunities to develop and sustain unique, innovative curriculum-driven programs that focus on various cancer education activities” [7]. In the two decades thereafter, this FOA was reissued six times to fund the R25 CEGP until 2015 [8]. During this period of time, as cancer research underwent a rapid expansion in scope, the R25 CEGP evolved concurrently to support education projects in a wide range of cancer research fields in the forms of Short-term Education (skills development) programs, Short-term Research Experience programs, and Institutional Curriculum Development projects [8].^1^

Since 2015, the R25 CEGP has undergone a transition by utilizing multiple specialized FOAs each funding a different form of R25 project. While using a single FOA to support various forms of educational projects afforded programmatic flexibility, it also presented a challenge for assessment of these programs as a whole because of the difficulty in comparing the merits of projects that differ in their goals, scopes, and activities. In an effort to enable evaluation of similar forms of R25 programs using comparable standards, NIH revised the guidelines for the R25 program to require the use of specialized R25 FOAs each supporting education projects involving the same type of activity(ies). Furthermore, the revised NIH R25 guidelines require designated FOAs for Programs to Promote Diversity, which provide funding for R25 programs focusing on enhancing the workforce diversity in biomedical, clinical, behavioral, and social sciences. This policy change led to the publication of five NCI R25 FOAs [9–13], collectively supporting the R25 CEGP previously funded by a single FOA [8]. This transition to individual FOAs also led to an administrative change. The NCI Cancer Training Branch in the Center for Cancer Training that solely administered the R25 CEGP in the past now shares this responsibility with the Diversity Training Branch in the Center to Reduce Cancer Health Disparities, which manages R25 FOAs for Programs to Promote Diversity.

The needs to adequately communicate with the cancer education community about the transition the R25 CEGP is currently undergoing, as well as to gather ideas to strengthen the program at a time when breakthroughs in cancer research are achieved at a breath-taking pace, led to NCI’s decision to sponsor the “NCI Cancer Education Workshop 2016” on September 13, 2016 in Bethesda, Maryland (Appendix 1). Fifty-three leaders in cancer education (Appendix 2), including R25 CEGP applicants, principal investigators (PIs), and peer-reviewers; NIH/NCI staff with leadership roles in the policy, program, and scientific review of the R25 CEGP; and representatives from professional organizations with active roles in cancer education participated in the workshop. This report is a summary of the proceedings of the workshop.

## Cancer Education Grant Program Overview

In the first session of the workshop, NCI staff provided an overview of the R25 CEGP. NCI has invested approximately $13 million in the R25 CEGP in each of the past 5 years. On average, NCI received 36 competing applications and awarded 11 new R25 grants per year, for an annual success rate of 30.5%. At the end of FY 2016, the R25 CEGP portfolio includes 52 active awards. The impact of the program is far reaching as these awards support educational activities in a broad range of cancer research fields benefiting more than 3200 CEGP participants at various career stages every year.

The five active FOAs (Table 1) supporting the R25 CEGP are: (i) PAR-15-150 (Curriculum or Methods Development), which supports the development of new curricula, novel instructional approaches or tools that are readily adaptable by the cancer education community; (ii) PAR-15-151 (Courses for Skills Development), which supports innovative graduate-level courses that increase the core skills and/or enhance the motivation of participants to consider a cancer-focused career; (iii) PAR-15-152 (Research Experiences), which supports innovative, hands-on, cancer research experiences to stimulate interest in further training in cancer research; (iv) PAR-16-138 (Program to Promote Diversity-Research Experiences); and (v) PAR-16-139 (Program to Promote Diversity-Courses for Skills Development). The last two FOAs are similar to PAR-15-152 and PAR-15-151, respectively, but are designated for participants who are underrepresented in biomedical, clinical, behavioral, and social sciences. Collectively, these five FOAs support the activities previously supported by a single FOA [8]. Each of the current FOAs has specific requirements/instructions on issues such as the eligible types of participants, allowable budget and budget structure, and the duration of the grants. Table 1 below summarizes the key features of the new FOAs. Full details can be found in the FOAs [9–13].

**Table 1:** Summary of the five current NCI R25 CEGP FOAs

	PAR-15-150 (Curriculum or Methods Development)	PAR-15-151 (Courses for Skills Development)	PAR-15-152 (Research Experiences)	PAR-16-138 (Program to Promote Diversity-Research Experiences)	PAR-16-139 (Program to Promote Diversity-Courses for Skills Development)
Award project period	Up to 3 years	Up to 5 years	Up to 5 years	Up to 5 years	Up to 5 years
Award budget^a^	Up to $100,000	Up to $300,000	Up to $300,000	Up to $300,000	Up to $300,000
Personnel (salary/fringe) and consultant costs	As appropriate	Up to $150,000	$3500 per participant	$3500 per participant	Up to $150,000
Participants	Not applicable	Researchers, healthcare providers, graduate and health professional students, certain lay community members	Undergraduate, graduate, and health professional students	Primarily underrepresented undergraduate, graduate, and health professional students, postdoctoral fellows, residents, and faculty	Primarily underrepresented undergraduate, graduate, and health professional students, researchers, and healthcare providers
Per participant costs	Not applicable	Subsistence: none	Subsistence: Up to $6000 (undergraduates); up to $7200 (graduate/health professional students)	Salary: Up to $6000 (undergraduates); up to $7200 (graduate/health professional students); up to $12,350 (postdoctoral fellow/residents/faculty)	Salary: none
		Materials: $500 Housing/per diem: $1000Travel costs^b^	Research costs: $1000 Housing: $1000Travel costs	Research costs: $1000 Housing: $1000Travel costs	Materials: $500 Housing/per/diem: $1000Travel to/from course
Competing renewals allowed?	No	Yes	Yes	Yes	Yes

^a^All costs listed are direct costs per year

^b^Travel costs allow one round-trip to/from the R25 program site for non-local participants

## Showcase of Currently Funded R25 CEGP Projects

In the next two sessions of the workshop, the PIs of six currently funded programs each gave a 30-min presentation to showcase the goals, designs, and accomplishments of their respective programs. These programs, some long-standing while others relatively new, feature various types of education activities that the R25 CEGP supports. Below is a summary of these presentations.

i. “Methods in Clinical Cancer Research Workshop,” (R25CA068647, PI: Daniel D. Von Hoff, American Association for Cancer Research) [14].

This program represents an example of a “Courses for Skills Development” type of R25 project. It is an intensive one-week educational workshop held annually in Vail, Colorado and partially supported by an R25 CEGP award since 1996. The goal of the workshop is to teach young investigators clinical trial design and implementation. Each summer, 100 early career oncology fellows and junior faculty participate in the workshop, taught by 50 dedicated faculty including clinical investigators, biostatisticians, and patient advocates. Each participant brings to the course a protocol concept, which he or she develops into an IRB-ready clinical trial protocol with associated informed consent documents during the week. A mix of didactic lectures, small group discussions, and one-on-one mentoring is combined with daily protocol development group sessions to provide training in the methods, design, and conduct of clinical trials. Follow-up questionnaires completed by participants between 1 and 5 years after the workshop are used to measure the impact of the workshop on the participants’ careers. To date, 2100 participants have completed the workshop, which represent a significant portion of the nation’s research oncologist community.

ii. “Integrated course in Biology and Physics of Radiation Oncology (IBPRO),” (R25CA171971, PIs: Michael C. Joiner and Monica Tracey, Wayne State University) [15].

This program is another example of the “Courses for Skills Development” type of R25 project. This 5.5-day program in advanced radiobiology and medical physics is designed to address the disconnection between the increasing demand for radiotherapy and the decreasing activity in radiation research. The course is organized around a set of pertinent themes in radiation oncology and includes both physics- and biology-oriented, theme-related keynote lectures, as well as interactive activities, such as a Virtual Hospital, analysis of radiation oncology protocols, panel discussions, debates of controversial topics in radiation oncology, and networking activities. The in-person course is enriched by additional materials provided online. Approximately 15 faculty members teach the course each year. Fifty participants are recruited annually among researchers and clinicians who either work or are interested in radiation oncology. Each year, at least three potential new faculty members are invited to participate and are provided with additional mentoring so that they may contribute to IBPRO in subsequent years and also build their own local training programs. Since 2013, over 140 individuals have participated in IBPRO, and their evaluations of the course have been gathered by surveys.

iii. “A National Curriculum in Cancer Genomics for Pathology Residents,” (R25CA168544, PI: Richard L. Haspel, Beth Israel Deaconess Medical Center) [16–18].

This is a combined “Curriculum Development” and “Courses for Skills Development” R25 project developed by the national Training Residents in Genomics (TRIG) Working Group. The objective of the curriculum is to provide pathologists the much needed knowledge and skills required to practice genomic medicine, focusing on next-generation sequencing techniques that allow molecular prognostic stratification of patients and genotype-guided therapy. The curricular resource includes readings, lectures, and team-based learning activities. Since 2013, the curriculum has been deployed in over 20 national and international workshops and courses, often held in conjunction with annual meetings of professional societies. Participants typically include pathology trainees and practicing pathologists but there have also been “train-the-trainer” sessions for geneticists. The evaluation of the national implementation and efficacy of the curriculum is based on results of the pathology resident in-service exam (RISE), which all US pathology residents complete as a test of competency. To facilitate dissemination of the curriculum, an instructor handbook and toolkit have been developed and downloaded from the TRIG website by over 450 participants. Online versions of the learning exercises have also been developed and are currently being evaluated in 10 residency programs.

iv. “UAB Cancer Research Experiences for Students,” (R25CA076023, PIs: John W. Waterbor and Peter Smith, University of Alabama at Birmingham (UAB)) [19–21].

The “UAB Cancer Research Experiences for Students” (CaRES) program, supported by an NCI R25 award since 1999, represents an example of the “Research Experiences” type of project. The goal of the program is to motivate participants to consider careers in cancer research by providing them with the opportunity to complete a cancer research project under the mentorship of a faculty preceptor that could result in a publication. CaRES participants include medical, veterinary, pharmacy, and graduate public health students from UAB and three other universities in Alabama. Forty participants complete an 8 to 12-week summer internship at UAB or the Hudson Alpha (HA) Institute for Biotechnology in Huntsville, AL. Students use a “self-matching” process to identify best-suited research projects available to them and complete a mentoring contract with a corresponding faculty preceptor drawn from approximately 400 UAB faculty members. In addition to their research projects, CaRES participants also propose an Individual Development Plan, attend a seminar series, take part in clinical “shadowing” opportunities, and present their research findings locally at a CaRES forum. Some students also present their research regionally and nationally, as well as publish the findings with their preceptors. To date, over 600 students, about 20% of them underrepresented individuals, have participated in CaRES. Longitudinal tracking of the CaRES graduates from 1999 to 2015 shows that 15% are actively involved in cancer research and 27% have published cancer-related, peer-reviewed papers.

v. “Cancer Prevention Education: Student Research Experiences,” (R25CA056452, PI: Shine Chang, University of Texas MD Anderson Cancer Center) [22, 23].

The objective of this “Research Experiences” project, supported by an NCI R25 award since 1992, is to encourage participants to consider careers in cancer prevention research. Each summer, 25 undergraduate, graduate, and health professional students are provided with a 10-week mentored cancer prevention research experience, along with career development opportunities, seminars, and other educational activities. Recruitment occurs nationally and through linkages with Texas Medical Center-affiliated academic institutions. The program emphasizes the cultivation and support of a pool of over 100 faculty mentors, and recognizes model mentors. The Program Leadership and Advisory Committee match prospective participants with mentors who have submitted descriptions of available research projects. At the conclusion of the R25 program, participants present their findings at two formal institutional events. Some participants then go on to present their research at professional meetings and publish with their mentors. To date, 364 students, approximately 29% of them underrepresented individuals, have completed the program. Longitudinal tracking of students who have participated in the program during the past 10 years shows that about half have completed their training, and of those, 53% are involved in research and 20% work in healthcare.

vi. “*Éxito!* Latino Cancer Research Leadership Training,” (R25CA134301, PI: Amelie G Ramirez, University of Texas Health Science Center at San Antonio) [24].

This combined “Courses for Skill Development” and “Research Experiences” type of R25 program focuses on doctoral-level preparation in the social, behavioral, and public health sciences for Latinos and intends to address the deficit of public health doctoral degrees awarded to Latinos. The program supports a five-day Summer Institute (SI) that is accompanied by web-based academic support, career-building opportunities, interactive online communication forums, and facilitated connections to pre- and post-doctoral programs. Each year, 25 participants are recruited nationally to take part in the SI, and up to 10 of them may subsequently be awarded a paid summer internship in Latino cancer control research that is also supported by the R25 award. Since 2010, 125 participants have attended an SI and report significantly greater confidence that they will apply to a doctoral program in the next 5 years, as well as significantly greater self-efficacy in their ability to be accepted to and succeed in a doctoral program. Of the SI participants from 2010 to 2015, 30% have applied to doctoral programs and 21% are currently enrolled in doctoral programs with the majority being interested in pursuing a cancer-related career.

## Group Discussions

Following the presentations, participants were assigned to four breakout groups for concurrent discussions of a set of broad-themed questions designed to gather ideas from the cancer education community to strengthen the R25 CEGP. Discussion leaders of each group then presented the salient points noted by their respective group to all participants for a general discussion. A synopsis of the discussion on each question is as follows:

i. What are the cancer research areas where R25 cancer education programs would make the greatest impact to the research/clinical workforce? Why?

A consensus high impact area for the R25 CEGP identified by all four breakout groups was to support cancer education programs that help bring a basic understanding of breakthroughs in emerging cancer research fields to a broad range of participants, including students, researchers, healthcare providers, and public health professionals. Examples of such emerging fields include cancer immunotherapy, precision medicine, genome editing/alteration technologies, as well as all types of “-omics” and “big data” science. Given that breakthroughs are being made at an unprecedented pace, R25-supported education programs offer an ideal means to help keep the cancer research/care communities updated with the progress. Another important area recognized was to continue to support projects that help healthcare providers better meet the needs of cancer patients as they transition from treatment to survivorship to palliative care and end of life, because the formal clinical training provided to healthcare providers in these areas remains inadequate. Among other suggestions were that the R25 CEGP should continue to support efforts to “train-the-trainers” through nurturing current program participants for a future faculty role or enhancing the teaching and mentoring skills of less experienced program faculty members; strengthen support for cancer education projects with international participation and/or learning activities; and encourage Research Experiences programs that provide participants with exposures beyond traditional research areas, such as community health, healthcare industry, and health policy.

ii. What are the most effective designs or models for R25 cancer education programs? What are their respective strengths?

Diversifying the R25 CEGP faculty was considered critical to strengthening the effectiveness of education programs. Traditionally, faculty members are predominantly academic researchers with expertise in fields related to the focus of a given R25 project. It was suggested that R25 education programs are ideal venues to recruit program faculty from industry, public health communities, patient advocacy organizations, and cancer patients and caregivers to teach alongside academic faculty. Such an approach would provide program participants with real world perspectives as well as exposure to multidisciplinary approaches and team science. With regard to new ways for R25 CEGP participants to acquire knowledge and skills, it was suggested that a team-based case study approach is highly effective as it offers excellent opportunities for participants with different backgrounds to learn from each other. Online and social media resources were recognized as effective tools for delivering education program content, providing mentoring, and conducting program evaluation. Furthermore, it was suggested that R25 education programs designed to enhance core professional competencies should consider awarding participants certificates or continuing education units (CEUs).

iii. Assuming that you are a member of the Study Section, what are the aspects of R25 cancer education programs that you think should be critically evaluated? Why?

R25 applications, like most other types of NIH grant applications, are evaluated primarily based on five review criteria, namely, significance, investigator(s), innovation, approach, and environment. Many participants pointed out that, because R25 CEGP applications collectively are expansive in scientific scope, diverse in methodologies, and varied in desired outcomes, it is crucial for applicants to clearly articulate the “significance” of the proposed program. Specifically, it was suggested that the inclusion of a thorough needs assessment supported by a literature review in the application is critical in establishing the “significance” of the proposed project. It was also noted that a clear description of appropriate methods and/or activities with measurable outcomes as benchmarks of success is essential for objective evaluation of an application’s “approach”. Furthermore, participants pointed out that diversity recruitment plans must include adequate descriptions of efforts to address the challenges a given program may encounter in recruiting underrepresented individuals, and competing renewal applications must accurately report the record of their diversity recruitment efforts during the prior period of grant support. There was also a very strong desire among workshop participants that all R25 CEGP applications should be reviewed appropriately and consistently. To that end, it is important that reviewers of CEGP applications fully understand the specific instructions for each review criterion as described in the FOAs and explained by NCI staff during the pre-review orientation.

iv. What are the most effective approaches for R25 cancer education programs, especially R25 Programs to Promote Diversity, to increase participation of underrepresented individuals and enhance workforce diversity?

Reaching out to Minority-Serving Institutions (MSIs) and professional societies, such as the National Medical Association (NMA) and the Society for the Advancement of Chicanos and Native Americans in Science (SACNAS), was identified as an effective strategy to increase the participation of underrepresented individuals in R25 cancer education programs. This strategy may also lead to collaborations between major academic institutions and MSIs/professional societies in developing new R25 programs. To this end, a number of workshop participants urged NCI to help leverage other NCI Programs to Promote Diversity such as the U54 Comprehensive Partnerships to Advance Cancer Health Equity (CPACHE), supported through PAR-15-103; the P20 Feasibility Studies to Build Collaborative Partnerships in Cancer Research, supported through PAR-16-084; and the Geographic Management of Cancer Health Disparities Program (GMaP) [25] to broaden the pool of underrepresented individuals for R25 cancer education programs. Another strategy noted by several participants was to raise the subsistence or salary provided to participants. It was pointed out that many underrepresented students need to maintain income streams for their families while participating in R25 cancer education programs, and paying a subsistence or salary that is at least equivalent to the local minimum wage may make it easier for these students to participate in R25 cancer education programs. Related to this point, it was suggested that allowing participation in R25 cancer research experience programs on a part-time basis might afford these students the flexibility to meet both their academic goals and personal commitments. Several other strategies, including recruiting diverse program faculty to serve as effective role models, demonstrating how cancer research helped improve health in underrepresented communities, and showcasing different types of cancer-related career options, were also considered effective in recruiting and retaining underrepresented students.

## Conclusions/Perspectives for the Future

The workshop succeeded in meeting all its stated objectives. NCI Program Staff provided an informative overview of the R25 CEGP, and a detailed explanation of the FOAs that support the program, which are of critical importance for the successful transition of the R25 CEGP. PIs of six current R25 programs, which feature diverse forms of education activities the R25 CEGP supports, showcased the successes of their respective programs. The in-depth discussion led to valuable suggestions to strengthen the R25 CEGP in meeting new cancer education needs brought about by rapid progress in cancer research, encouraging innovative educational approaches, improving R25 CEGP application preparation and peer review, and employing more effective strategies for enhancing diversity. One gratifying outcome of the workshop was that it brought together for the first time cancer educators who conduct different forms of education activities in different fields. The participants, including those who had engaged in cancer education for several decades, were pleasantly surprised that there were so many educators in the workshop whom they did not previously know. The excitement of being exposed to novel ideas and meeting new colleagues was evident at the gathering, and the workshop has already led to the initiation of several collaborations. There was a clear consensus that the NCI R25 CEGP offers a powerful platform for developing a stronger cancer education community. Continued efforts such as holding similar workshops periodically in the future will generate synergy among educators currently in the community and help bring many more dedicated educators into the community.

## Electronic supplementary material

ESM 1 (DOCX 45 kb)

ESM 2 (DOCX 45 kb)

## Footnotes

1 NCI also used the R25 funding mechanism to support the Cancer Education and Career Development Program, often known as the R25T program. This program has ended and merged into the NRSA T32 Training Grant Program, as announced in NOT-CA-13-008. In addition, NCI supports an R25 Youth Enjoy Science Program through PAR-17-059 that engages underrepresented students from grades 6 through college and their teachers. These R25 programs, however, are different from the R25 CEGP.
