# Supplementary material for: The National Cancer Institute R25 Cancer Education Grants Program: A Workshop Report
Source: J Cancer Educ. 2017 Jan 7;32(1):3–10. doi: 10.1007/s13187-016-1161-8 (PMC5290060; doi:10.1007/s13187-016-1161-8)
Supplement: Supplementary file 1 — (DOCX 45 kb) [file 13187_2016_1161_MOESM1_ESM.docx]

**Appendix 1: NCI Cancer Education Workshop 2016 Agenda – September 13, 2016.**

| 7:30-8:00 AM | Sign In / Pick Up Packets / Order Lunch | Ms. Yvetta Lewis |
| --- | --- | --- |
| 8:00-8:10 AM | Welcome / Opening Remarks | Dr. Jonathan Wiest NCI Center for Cancer Training |
| 8:10-8:15 AM | Logistics | Dr. Jeannette Korczak NCI Cancer Training Branch |
| **Session 1** | **Overview and Introduction Moderator: Dr. Jeannette Korczak** |  |
| 8:15-8:30 AM | Overview of NCI’s Cancer Education Funding Program | Dr. Ming Lei NCI Cancer Training Branch |
| 8:30-9:00 AM | Introduction of NCI R25 Funding Opportunity Announcements (FOAs) | Dr. Jeannette Korczak NCI Cancer Training Branch |
| 9:00-9:15 AM | Introduction of NCI R25 Programs to Promote Diversity FOAs | Dr. Davyd Chung NCI Diversity Training Branch |
| 9:15-9:30 AM | Break |  |
| **Session 2** | **Courses and Curriculum Development Programs Moderator: Dr. Jeannette Korczak** |  |
| 9:30-10:00 AM | Methods in Clinical Cancer Research Workshop | Dr. Daniel Von Hoff American Association for Cancer Research |
| 10:00-10:30 AM | Integrated Course in Biology and Physics of Radiation Oncology (IBPRO) | Dr. Michael Joiner Wayne State University |
| 10:30-11:00 AM | A National Curriculum in Cancer Genomics for Pathology Residents | Dr. Richard Haspel Beth Israel Deaconess Medical Center |
| 11:00-11:15 AM | Break |  |
| **Session 3** | **Research Experience and Diversity Programs Moderator: Dr. Davyd Chung** |  |
| 11:15-11:45 AM | UAB Cancer Research Experiences for Students | Dr. John Waterbor University of Alabama at Birmingham |
| 11:45-12:15 PM | Cancer Prevention Education: Student Research Experiences | Dr. Shine Chang University of Texas MD Anderson Cancer Center |
| 12:15-12:45 PM | *Éxito!* Latino Cancer Research Leadership Training | Dr. Amelie Ramirez University of Texas Health Science Center |
| 12:45-1:30PM | Lunch Break |  |
| **Session 4** | **Breakout Sessions** |  |
| 1:30-2:45 PM | Discussions will focus on education program models, research areas where education programs can impact the most, R25 grant review, and enhancing diversity through R25 programs. |  |
| 2:45-3:00 PM | Break |  |
| **Session 5** | **Reporting and Summary Moderator: Dr. Ming Lei** |  |
| 3:00-3:30 PM | Reporting by breakout groups |  |
| 3:30-4:15 PM | General Discussion |  |
| 4:15-4:30 PM | Summary |  |
| 4:30 PM | Meeting Adjourn |  |
