# Supplementary material for: The National Cancer Institute R25 Cancer Education Grants Program: A Workshop Report
Source: J Cancer Educ. 2017 Jan 7;32(1):3–10. doi: 10.1007/s13187-016-1161-8 (PMC5290060; doi:10.1007/s13187-016-1161-8)
Supplement: Supplementary file 2 — (DOCX 45 kb) [file 13187_2016_1161_MOESM2_ESM.docx]

**Appendix 2: List of Workshop Participants**

| **Name** | **Affiliation** |
| --- | --- |
| Amy Baran, Ph.D. | American Association for Cancer Research |
| Maria Bishop, M.D. | University of Arizona |
| Amy Bouton, Ph.D. | University of Virginia School of Medicine |
| William Breitbart, M.D. | Memorial Sloan Kettering Cancer Center |
| Jason Bush, Ph.D. | California State University, Fresno |
| Robert Chamberlain, Ph.D. | University of Nebraska Medical Center |
| Shine Chang, Ph.D. | University of Texas MD Anderson Cancer Center |
| Davyd Chung, Ph.D. | National Cancer Institute, National Institutes of Health |
| Melany Cueva, R.N., E.D.D. | Alaska Native Tribal Health Consortium |
| Betty Ferrell, Ph.D. | City of Hope |
| Heather Gerhart, M.A. | Keystone Symposia on Molecular and Cellular Biology |
| Ramaswamy Govindan, M.D. | Washington University School of Medicine |
| Richard Haspel, M.D., Ph.D. | Beth Israel Deaconess Medical Center |
| Bret Hassel, Ph.D. | University of Maryland School of Medicine |
| David Hein, Ph.D. | University of Louisville |
| Kathleen Heneghan, Ph.D., R.N. | American College of Surgeons |
| Chindo Hicks, Ph.D. | Louisiana State University Health Sciences Center New Orleans |
| Michael Joiner, Ph.D. | Wayne State University |
| Flora Katz, Ph.D. | Fogarty International Center, National Institutes of Health |
| Karen Kim, M.D., M.S. | University of Chicago |
| Wahid Al Kharusi, Mb.Ch.B. | Omani Ministry of Foreign Affairs, Sultanate of Oman |
| Jeannette Korczak, Ph.D. | National Cancer Institute, National Institutes of Health |
| Ming Lei, Ph.D. | National Cancer Institute, National Institutes of Health |
| Matthew Loscalzo, L.C.S.W. | City of Hope |
| Kay Lund, Ph.D. | Office of Extramural Research, National Institutes of Health |
| Grace Ma, Ph.D. | Temple University |
| Cathy Meade, Ph.D., R.N. | Moffitt Cancer Center |
| Tim Meeker, M.D., Ph.D. | National Cancer Institute, National Institutes of Health |
| Arthur Michalek, Ph.D. | University of Buffalo |
| Guy Montgomery, Ph.D. | Icahn School of Medicine at Mount Sinai |
| Charles Moore, M.D. | Emory/Healing Community Center |
| Folakemi Odedina, Ph.D. | University of Florida Gainesville |
| Peter Ogunbiyi, D.V.M., Ph.D. | National Cancer Institute, National Institutes of Health |
| Michael Ortiz, M.D. | Memorial Sloan Kettering Cancer Center |
| Rena Pasick, Dr.P.H. | University of California San Francisco School of Medicine |
| Susan Perkins, Ph.D. | National Cancer Institute, National Institutes of Health |
| Mark Pfeifer, M.D. | University of Louisville |
| William Pirl, M.D., M.P.H. | Sylvester Comprehensive Cancer Center, University of Miami |
| Mandi Pratt-Chapman, M.A. | George Washington University Cancer Institute |
| Amelie Ramirez, Dr.P.H. | University of Texas Health Science Center at San Antonio |
| William Redd, Ph.D. | Icahn School of Medicine at Mount Sinai |
| Erica Rosemond, Ph.D. | National Center for Advancing Translational Sciences, National Institutes of Health |
| Georgia Sadler, Ph.D., M.B.A. | University of California San Diego Moores Cancer Center |
| Kathleen Scotto, Ph.D. | Rutgers, The State University of New Jersey |
| Calvin Simerly, Ph.D. | University of Pittsburgh |
| Amr Soliman, M.D., Ph.D. | University of Nebraska Medical Center |
| Srinivas Sridhar, Ph.D. | Northeastern University and Harvard Medical School |
| LaMonica Stewart, Ph.D. | Meharry Medical College |
| Dhiraj Vattem, Ph.D. | Ohio University |
| Daniel Von Hoff, M.D. | American Association for Cancer Research and Translational Genomics Research Institute |
| John Waterbor, M.D., Dr.P.H. | University of Alabama at Birmingham (UAB) School of Public Health |
| Jonathan Wiest, Ph.D. | National Cancer Institute, National Institutes of Health (NCI/NIH) |
| Gerard Zambetti, Ph.D. | St. Jude Children’s Research Hospital |
